# Will EGFRvIII and neuronal-derived EGFR be targets for imipramine?

**DOI:** 10.3389/fphar.2023.1156492

**Published:** 2023-05-30

**Authors:** Zesheng Li, Bo Wang, Jianjian Wu, Lei Han

**Affiliations:** ^1^ Tianjin Neurological Institute, Key Laboratory of Post-Neuroinjury Neuro-repair and Regeneration in Central Nervous System, Ministry of Education and Tianjin City, Tianjin Medical University General Hospital, Tianjin, China; ^2^ Department of Environment, College of Environment and Resources, Xiangtan University, Xiangtan, China

**Keywords:** glioblastoma, tricyclic antidepressant (TCA), cancer biology, neuroscience < neuropharmacology, translational medicine, EGFR

## Abstract

Tricyclic antidepressant is an old and well-established therapeutic agent with a good safety profile, making them an excellent candidate for repurposing. In light of the growing understanding of the importance of nerves in the development and progression of cancer, attention is now being turned to using nerve-targeting drugs for the treatment of cancer, particularly TCAs. However, the specific mechanism by which antidepressants affect the tumor microenvironment of glioblastoma (GBM) is still unclear. Here, we combined bulk RNA sequencing, network pharmacology, single-cell sequencing, molecular docking and molecular dynamics simulation to explore the potential molecular mechanism of imipramine in the treatment of GBM. We first revealed that the imipramine treatment is presumed to target EGFRvIII and neuronal-derived EGFR, which may play a pivotal role in treating GBM by reducing the GABAergic synapse and vesicle-mediated release and other processes thereby modulating immune function. The novel pharmacological mechanisms might provide further research directions.

## Introduction

Glioblastoma (GBM) is known as the most aggressive type of intracranial tumor. High morbidity and recurrence rates represent poor prognosis in GBM patients. Treatments involving monotherapy are often not effective enough to suppress GBM tumor cells. First-line treatment of GBM patients is usually a combination therapy, including maximal safe surgical resection, postoperative radiotherapy, and chemotherapy with temozolomide. Although advances have been made in the field of surgery, radiotherapy, and chemotherapy, the median survival time (15–16 months) for GBM patients remains below expectations (Ostrom et al., 2018). Thus, novel therapeutic approaches for improving the survival of GBM patients are urgently needed.

Tricyclic antidepressants (TCAs) were originally developed and marketed for the treatment of depression, but have been used to treat a wide range of conditions, most commonly off-label. Long-term use of TCAs is associated with a lower incidence of gliomas (Walker et al., 2011). A previous study found that imipramine, a TCA, increased autophagy and resulted in therapeutic benefits in animals bearing GBM tumors (Shchors et al., 2015). Bielecka-Wajdman et al. reported that several antidepressants, including imipramine and amitriptyline, promoted the conversion of glioma stem cells to non-glioma stem cells, thereby reversing the malignant phenotype (Bielecka-Wajdman et al., 2017). Recently, Chryplewicz et al. describe imipramine increases autophagic flux within cancer cells, thereby recruiting T cells, as well as reprogramming macrophages as pro-inflammatory cells by inhibiting the histamine receptor on the membrane (Chryplewicz et al., 2022). Considering the psychological pressure of GBM patients, as well as the resulting anxiety and depression, using imipramine to treat GBM patients can kill two birds with one stone. Since depression is a frequent occurrence in GBM patients as well as there are overlaps between molecular and cellular mechanisms involved in both diseases’ pathogenesis, antidepressants that act as antitumor agents are an attractive treatment option for GBM patients (Abadi et al., 2022). However, how imipramine regulates crosstalk between various cells in the GBM microenvironment deserves further exploration.

As a strategy, network pharmacology is a powerful tool for examining the complex mechanisms that cause disease and the effects of drugs. Molecular Docking is the process of predicting the structure of molecules using computer modeling. This method is widely used to discover new medicines and mechanisms of action for pharmaceuticals (Pinzi and Rastelli, 2019). In this study, we aim to use bulk RNA sequencing, network pharmacology, single-cell RNA-seq analysis, molecular docking and molecular dynamics simulation to explore the mechanism of imipramine in treating GBM. We uncovered the crucial role of imipramine in GABA (Gamma-aminobutyric acid) and immune function regulation in GBM patients. We hypothesize that imipramine may target neuronal cell-derived EGFR, thereby modulating the immune microenvironment. We also found that imipramine has a strong binding ability to EGFRvIII, which may provide a new potential drug for clinical treatment.

## Methods

### Dataset collection and preprocessing

RNA-seq data from GSE4290 was downloaded from Gene Expression Omnibus (GEO, https://www.ncbi.nlm.nih.gov/gds) database. The screening criteria were as follows: First, the dataset including GBM samples and non-tumor samples. Second, both non-tumor samples and GBM samples should be greater than 20 to ensure the quality of WGCNA. The GSE4290 dataset includes 81 samples from patients with GBM as well as 23 normal samples from healthy individuals (Sun et al., 2006). After preprocessing, we finally obtained 77 GBM samples and 23 normal samples with clinical data.

### Obtaining targets for imipramine

The detailed information of imipramine was obtained from PubChem (https://pubchem.ncbi.nlm.nih.gov/). The SwissTargetPrediction database (http://www.swisstargetprediction.ch/) (Daina et al., 2019) and the TargetNet database (http://targetnet.scbdd.com/home/index/) (Yao et al., 2016) as well as CTD (https://ctdbase.org/) (Davis et al., 2023), and BindingDB (https://bindingdb.org/bind) (Chen et al., 2002) were utilized for identifying potential target genes. A further search was conducted to access target proteins names using the UniProt database (https://www.uniprot.org/uploadlists/).

### Gene Ontology and pathway analysis

Gene Ontology (GO) and Kyoto Encyclopedia of Genes and Genomes (KEGG) pathway analysis was performed by clusterProfiler R package with a threshold of adjusted *p*-value <0.05 (Wu et al., 2021).

### Identification of DEGs

The R package “limma” was used to identify the differentially expressed genes (DEGs) between the GBM samples and healthy controls (Ritchie et al., 2015). |log2 (foldchange)| > 2 and the adjusted *p*-value <0.05 were used as screening criteria.

### Gene set enrichment analysis

GSEA is used to determine whether a defined set of genes shows a statistically significant difference in two different traits. Pathways that may play a role in the pathogenesis of GBM were explored using GSEA. The R package clusterProfiler was used for GSEA analysis. The following settings were used for the analysis: false discovery rate (FDR) < 0.25, adjusted *p*-value <0.05, |normalized enrichment score (NES)| > 1, which to be considered significant.

### WGCNA analysis of GEO

All genes were selected to construct the weighted gene co-expression network. First, a hierarchical clustering analysis was conducted to exclude the outlier samples. The Pearson correlation value was computed for each pair of genes. Then, an adjacency matrix was conducted. The WGCNA package’s “pickSoftThreshold” function was utilized to select the optimal β that satisfies the scale-free distribution (Langfelder and Horvath, 2008). The adjacency matrix was converted into two matrices: the topological overlap matrix (TOM) and 1-TOM. The TOM reflects the similarity between genes, while 1-TOM reflects the dissimilarity among them. Last, hierarchical clustering was used to classify genes into distinct modules. The module eigengene (ME) was computed to reflecting the gene expression profiles for each module. The settings of parameters were as follows. The soft threshold β = 6, minModuleSize = 50, mergeCutHeight = 0.25.

### Generation of protein-protein interaction (PPI) networks

Screened genes were analyzed in STRING (version 11.5, https://string-db.org/) to investigate protein-protein interactions (PPI) (Szklarczyk et al., 2019). PPI was constructed and visualized using Cytoscape software (version 3.9.1) (Shannon et al., 2003). A Molecular Complex Detection algorithm (MCODE) was used to detect dense regions of tightly bound proteins or PPI. The MCODE algorithm is used to identify critical sub-networks that contribute to the development of GBM and to identify critical subpopulation genes. The parameters of MCODE were all set using default settings.

### Single-cell RNA sequencing data analysis

TISCH2 is a database that utilizes single-cell RNA sequencing (scRNA-seq) technology to concentrate on the tumor microenvironment (TME) and its immune response against tumors (Han et al., 2023).

For a single gene, TISCH2 supports exploring its expression level across different single-cell datasets or cancer types. We explored the expression level of EGFR gene in different single-cell datasets with TISCH2.

The scTIME Portal is a database and a portal for single cell transcriptomes of tumor immune microenvironment (Hong et al., 2021). We employed it to obtain the expression of pathway genes in all cell types in GSE84465. We also used scTIME Portal to perform the cell-to-cell communication analysis in GSE84465.

### Analysis related to wild-type EGFR and mutant EGFR

CAMOIP is a tool for analyzing the expression data and mutation data from the TCGA. All analyzes related to wild-type EGFR and mutant EGFR were performed on CAMOIP (http://www.camoip.net/) (Lin et al., 2022).

### Molecular docking and molecular dynamics simulation

Firstly, the protein was optimized before docking using the Protein Preparation Wizard, and the ligand was prepared with LigPrep tool. We utilized the OPLS-2005 force field to provide partial atomic charge attribution, protonation states generation, and energy minimization. Grid-Based Ligand Docking with Energetics (Glide v11.5, Schrödinger) was used to dock all ligands into the catalytic pocket of the RPT protein in “extra precision mode” without applying any constraints. Finally, the complex with the lowest score was selected for further study. The molecular dynamics simulation protocol can be found in previous article (Wu et al., 2023).

### Statistical analysis

R software (version 4.2.0) (http://www.r-project.org) was used for statistical analysis. Wilcoxon tests were used to analyze subgroup differences. *p* < 0.05 was used as the threshold for statistical significance.

## Result

### Target genes for imipramine

**FIGURE 1:**
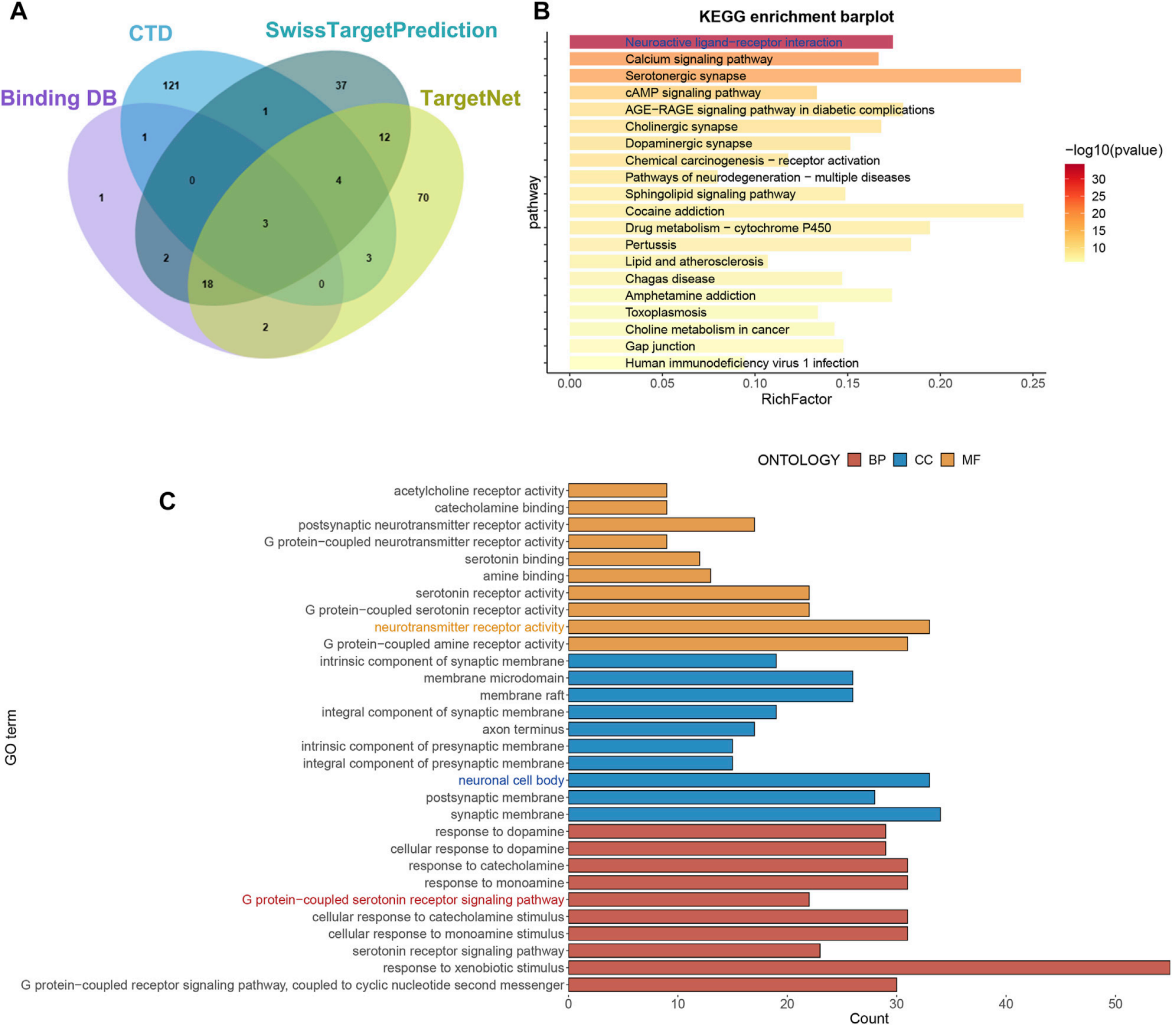
Obtain drug targets and functional analysis. **(A)** The drug targets were obtained from four databases. **(B)** KEGG analysis of drug targets. **(C)** GO analysis of drug targets.

The SwissTargetPrediction, CTD, BindingDB, and TargetNet databases were used to identify target genes associated with imipramine. Finally, 275 target genes associated with imipramine were identified and retrieved from these databases (Figure 1A; Supplementary Table S1). KEGG enrichment analysis shows that drug target genes were mainly enriched in neuroactive ligand−receptor interaction, serotonergic synapse, and calcium signaling pathway (Figure 1B). GO enrichment analysis shows that the enriched pathways were mainly involved in BP: response to xenobiotic stimulus, cellular response to catecholamine stimulus; CC: synaptic membrane, neuronal cell body; MF: neurotransmitter receptor activity, G protein−coupled amine receptor activity (Figure 1C). In total, the enrichment results showed that imipramine might be affecting neurotransmitter signaling circuits.

### Identification of DEGs between GBM and control

**FIGURE 2:**
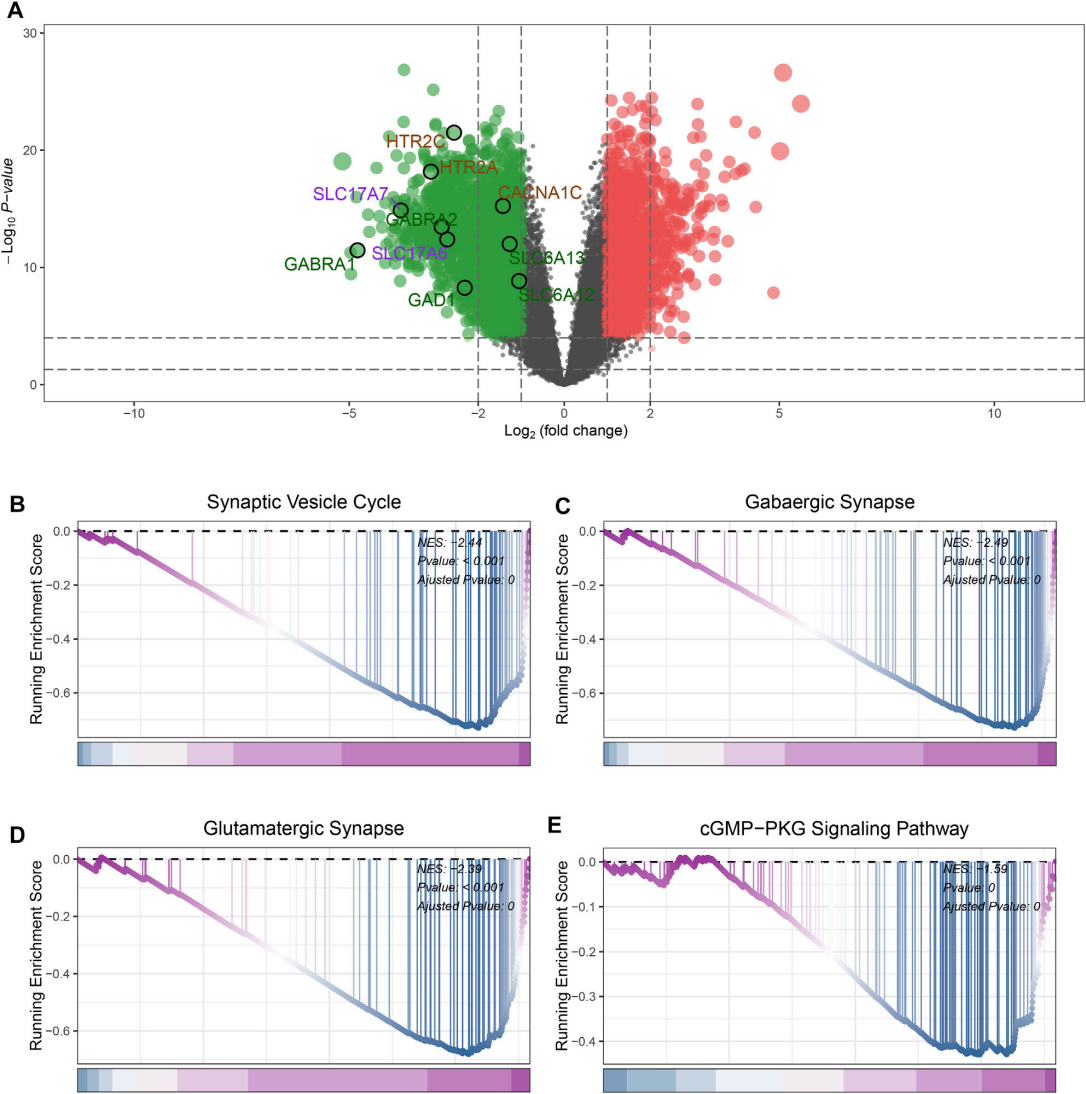
Identification of DEGs and GSEA analysis. **(A)** The volcano plot of DEGs expression. **(B)** GSEA results of Synaptic Vesicle Cycle pathway. **(C)** GSEA results of GABAergic Synapse pathway. **(D)** GSEA results of Glutamatergic Synapse pathway. **(E)** GSEA results of cGMP-PKG Signaling Pathway.

In GSE4290 datasets, first, we removed the batch effect between the samples (Supplementary Figure S1). According to the filtering criteria (|log2 (foldchange)| > 2 and the adjusted *p*-value <0.05), a total of 777 DEGs were screened, of which 207 DEGs were upregulated and 570 DEGs were downregulated (Supplementary Table S2). The result of the differential analysis was visualized in Figure 2A. Some genes participated in the pathways including Gabaergic Synapse, Synaptic Vesicle Cycle were significantly downregulated in GBM samples (Figures 2A–C). This phenomenon is consistent with previous reports (Jung et al., 2019).

### GSEA analysis

GSEA analysis was performed to investigate the differences in pathway landscapes between GBM and normal samples. We noticed that Synaptic Vesicle Cycle, GABAergic synapse, Glutamatergic Synapse and cGMP−PKG Signaling Pathway were significantly downregulated in GBM group (Figures 2B–E). These results indicate synthesis and vesicle-mediated release of the neurotransmitter GABA as well as signal transduction activities may be closely related to the pathogenesis of GBM.

### Construction of the weighted gene co-expression network

**FIGURE 3:**
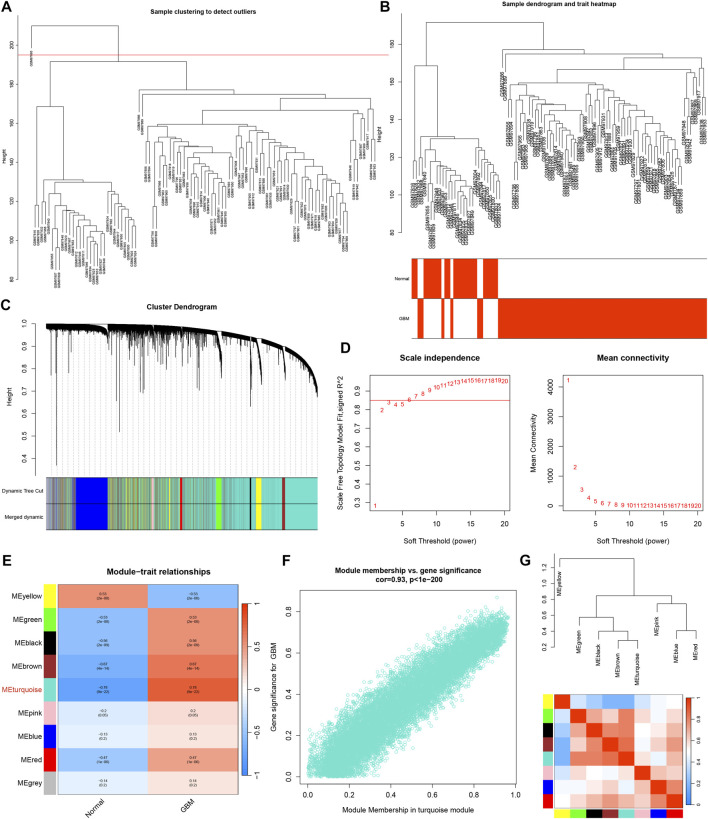
WGCNA analysis. **(A)** Raw cluster tree. **(B)** Sample dendrogram and trait heatmap. **(C)** The cluster dendrogram of WGCNA. **(D)** The soft threshold power and mean connectivity of WGCNA. **(E)** Correlations between gene modules and GBM status. **(F)** The correlation between the turquoise module memberships and the gene significance. **(G)** The correlation between modules.

The GBM samples and healthy control subjects from the GSE4290 dataset were analyzed utilizing the WGCNA package in R software. The outlier samples were removed (Figure 3A). The sample dendrogram and trait heatmap was shown in Figure 3B. The cluster dendrogram was shown in Figure 3C. The matrix was transformed into an adjacency matrix using the soft-thresholding parameter, denoted as β. The *R* ^2^ achieved more than 0.85 when β = 6, which became the power of our adjacency matrix (Figure 3D). After merging modules with similarity >0.75, nine co-expression modules were identified using the hierarchical clustering method of which the turquoise module exhibits the highest correlation with GBM (R = 0.78, *p* < 0.001), as shown in Figure 3E. Meanwhile, the scatter plot of MM relative to GS showed a good correlation between GS and MM within the turquoise module (R = 0.93, *p* < 0.001) (Figure 3F). Thus, the turquoise module could be a pivotal module closely associated with GBM. Additionally, the network heatmap was shown in Figure 3G.

### Functional enrichment analysis

**FIGURE 4:**
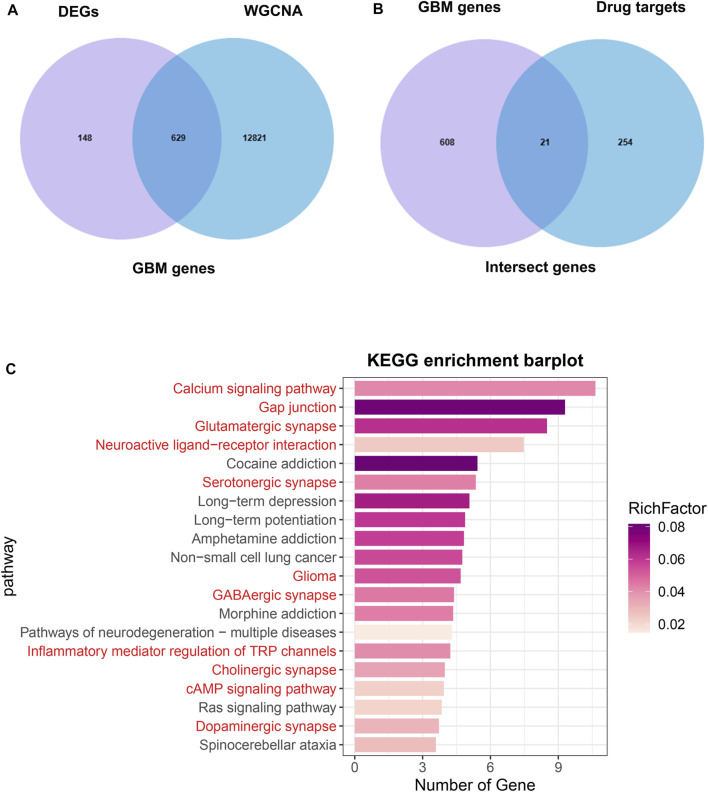
Identification of intersect genes and functional analysis. **(A)** The intersection of DEGs and WGCNA turquoise module genes were named GBM genes. **(B)** The intersection of GBM genes and drug targets genes were named intersect genes. **(C)** KEGG analysis of intersect genes.

The overlap between the DEGs and hub genes in the turquoise module was shown in Figure 4A, which was named GBM genes (Supplementary Table S3). The overlap between the GBM genes and Drug targets was shown in Figure 4B, which was named intersect genes. Then, we performed KEGG analysis on intersect genes. KEGG enrichment analysis showed that the enriched pathways were also mainly involved in Calcium signaling pathway, Gap junction, Glutamatergic synapse, Neuroactive ligand−receptor interaction, Serotonergic synapse, Glioma, GABAergic synapse (Figure 4C).

### Selection of key target genes

It is widely recognized that drugs elicit diverse effects on their target genes or target proteins. These effects may encompass the inhibition or upregulation of target gene expression, as well as the enhancement or suppression of target protein activity. Importantly, the precise regulatory effect of a drug on its target requires experimental validation. In this study, we solely analyze and predict the regulatory impact based on existing literature reports, with the aim of offering theoretical insights for subsequent researchers.

Traditional network pharmacology articles construct PPI with the intersect genes of drug targets and disease-related targets. However, this approach may not be entirely accurate, because there is a regulatory effect between proteins, and the drug targeting its own target protein may also affect the activity of other proteins. So, we do not only focus on the intersect genes and we selected all drug target genes and GBM genes to perform a large-scale PPI network and screen the key cluster with most interactions to explore the regulatory relationship between drug targets and disease-related targets.

**FIGURE 5:**
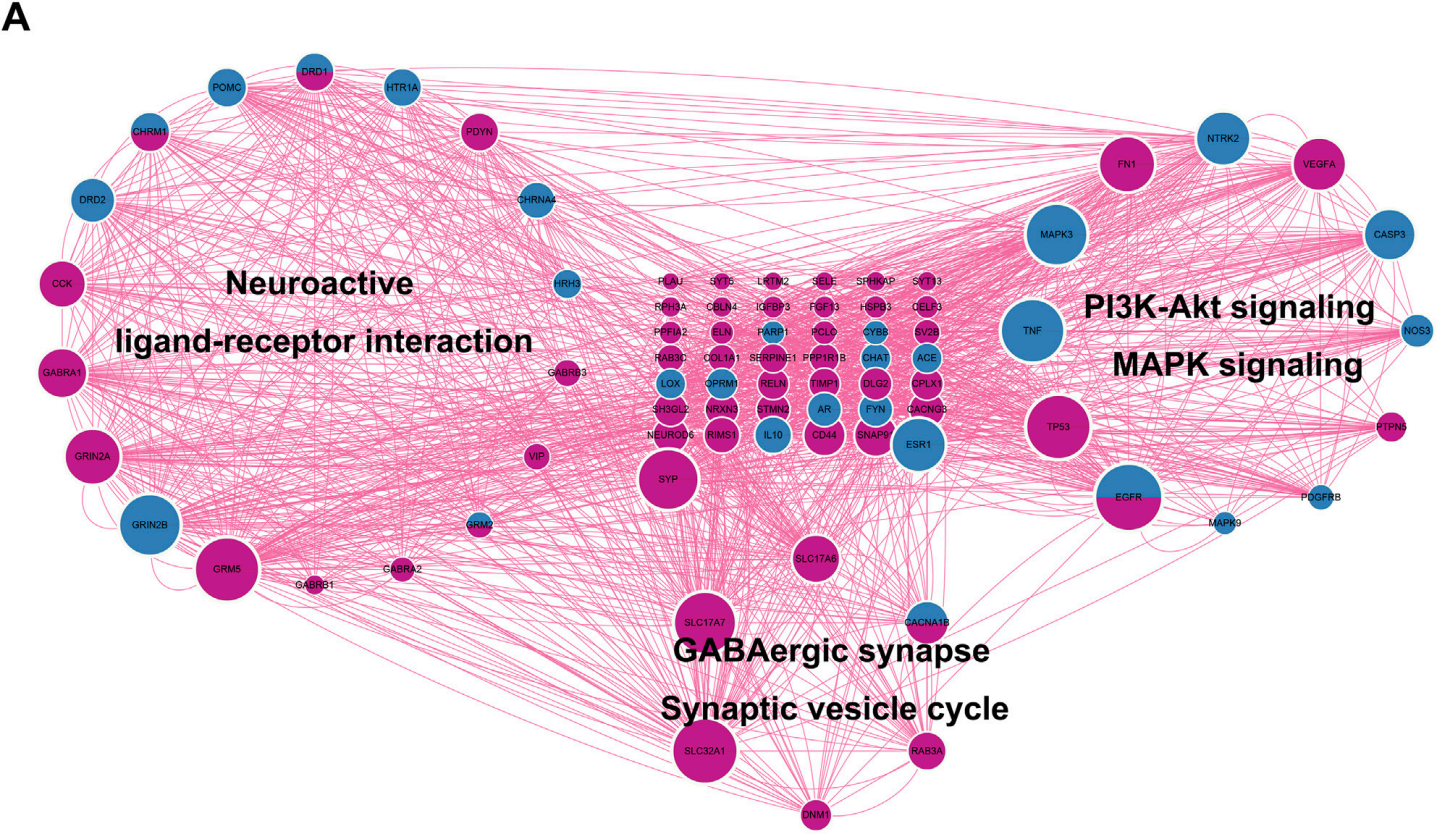
Identification of key cluster proteins and functional analysis. **(A)** The PPI network of key cluster proteins. DeepPink: GBM genes. Blue: drug targets genes. DeepPink and blue: both GBM gene and drug target.

A total of 883 drug target genes and GBM genes were imported into the STRING online database (version 11.5) to construct the PPI network. Then, the key cluster proteins were identified by Cytoscape’s plugin code “MCODE”. The key cluster proteins with the most interactions were visualized in Figure 5A. It is not difficult to infer that imipramine affects the progression of GBM by regulating the release and transmission of the neurotransmitter, such as GABA, thereby affecting MAPK signaling and PI3K-AKT signaling. In Figure 5A, the size of protein represents the degree of the protein, we noticed that EGFR is both a drug target and a GBM gene, while also exhibits a high degree. So, we infer EGFR as a key target for imipramine, which plays important role in MAPK signaling, PI3K-AKT signaling, Neuroactive ligand−receptor interaction, Synaptic Vesicle Cycle and GABAergic synapse.

### Single-cell analysis

**FIGURE 6:**
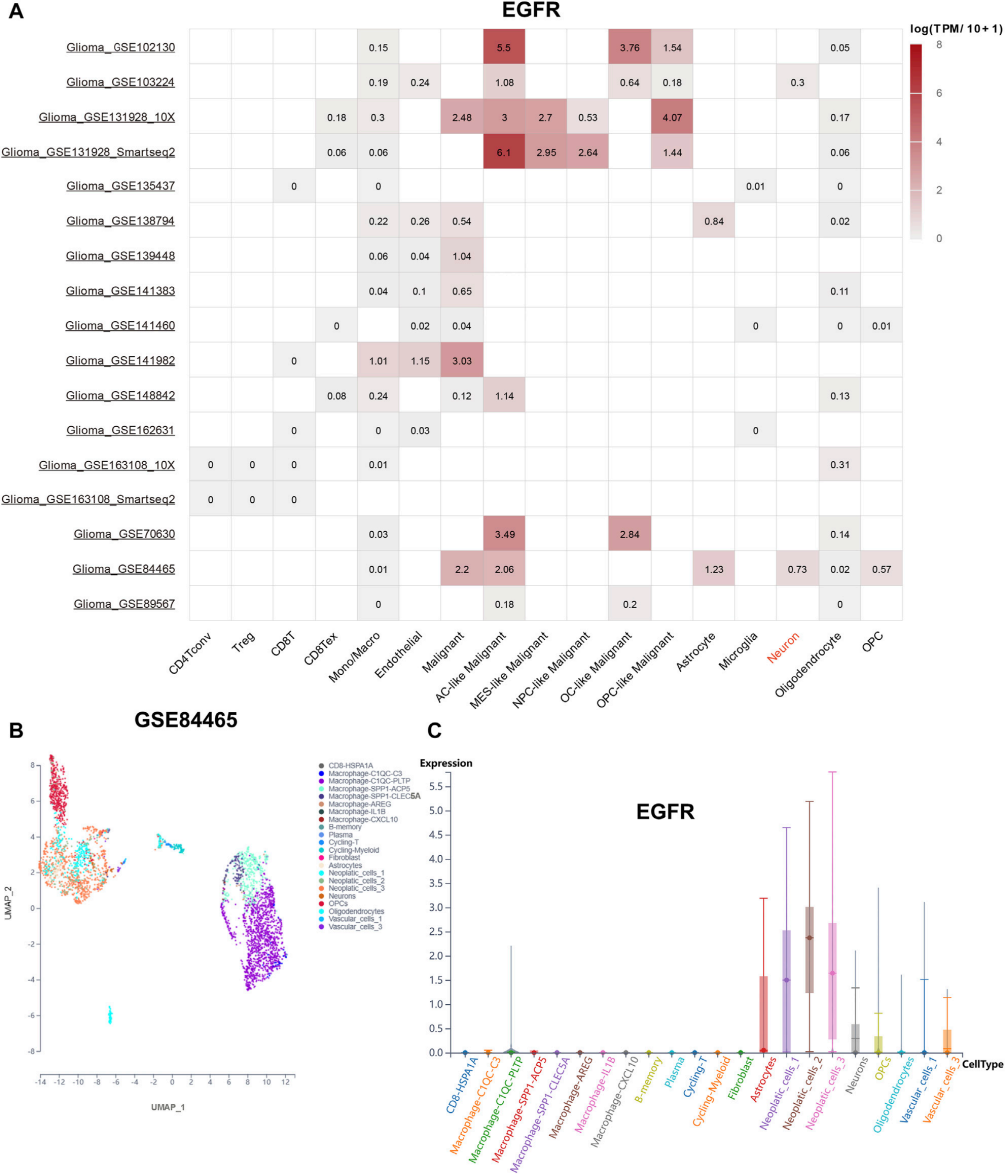
EGFR distribution landscape. **(A)** the distribution of EGFR in the different single-cell datasets. **(B)** the UMAP plot of GSE84465. **(C)** the distribution of EGFR in GSE84465.

To explore the distribution of EGFR genes in tumor microenvironment (TME), online datasets TISCH2 was utilized. The expression level of EGFR in different cells was shown in Figure 6A. Interestingly, we noticed that EGFR was expressed on neurons with a relative high level in GSE84465. In this way, the GBM single-cell RNA-seq dataset GSE84465 was chosen for further analysis. The Uniform Manifold Approximation and Projection (UMAP) plot of GSE84465 was shown in Figure 6B. The distribution of EGFR in different cell types was shown in Figure 6C. We can see that EGFR was expressed in neurons and astrocytes, implicating its potential role in the regulation of neurotransmitter.

**FIGURE 7:**
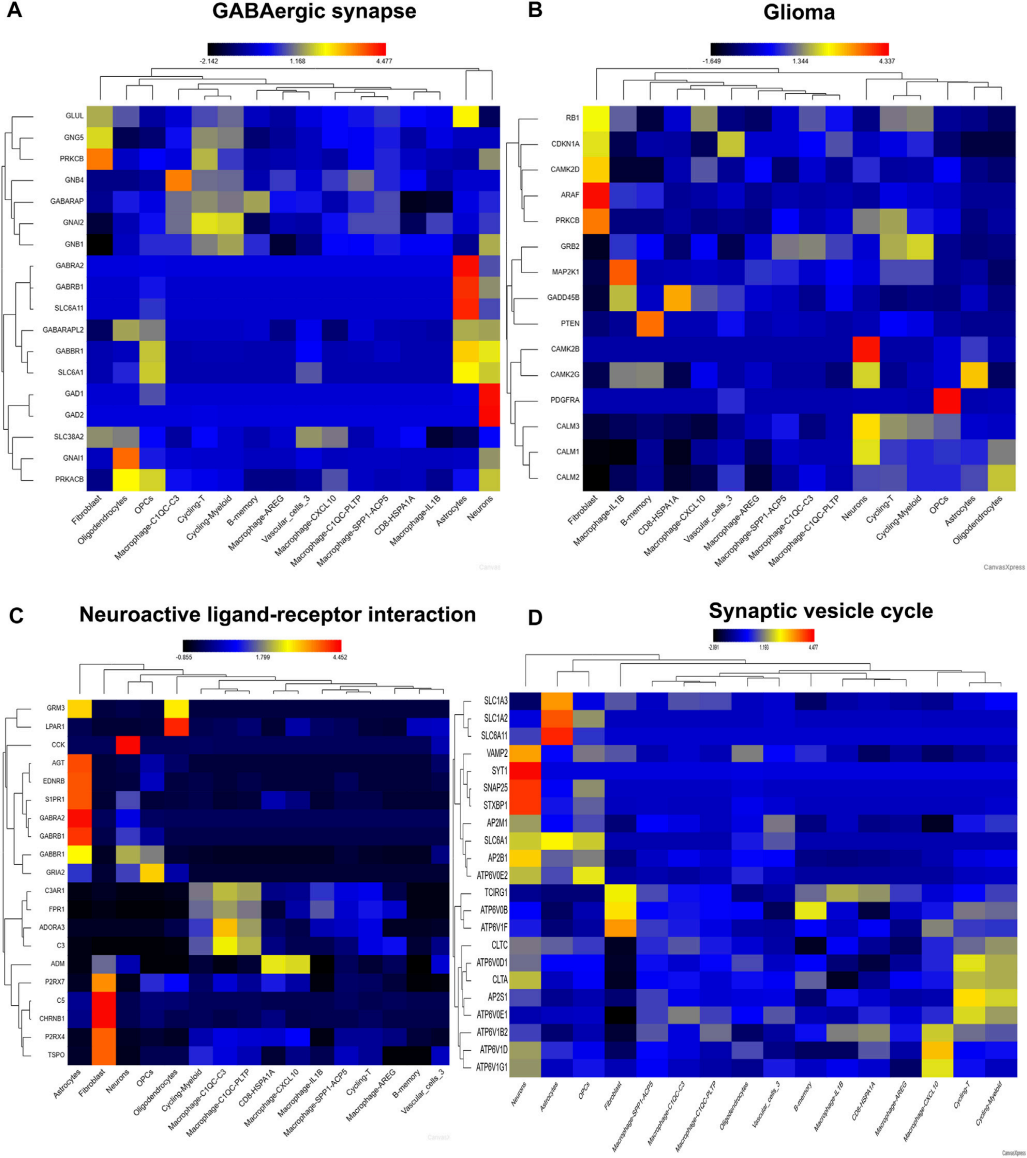
The distribution of pathway signatures in GSE84465. **(A)** GABAergic synapse pathway **(B)** Glioma pathway **(C)** Neuroactive ligand-receptor interaction pathway **(D)** Synaptic vesicle cycle pathway.

Then, we visualize four pathways involving in neurotransmitter activity (Figures 7A–D). Genes involving in GABAergic synapse mainly distributed in fibroblast, neurons, astrocytes, cycling-T, cycling-Myeloid, macrophage, implicating these genes may play important role in immune cell fate (Figure 7A). Genes involving in Glioma pathway were also distributed in neurons, fibroblast, and macrophage, B-memory, which confirmed the importance of neurons, stromal cells and immune cell in the pathogenesis of glioma (Figure 7B). Consistently, the genes involving in Neuroactive ligand−receptor interaction were also distributed in astrocytes, neurons, fibroblast, macrophage and CD8 cells (Figure 7C). Genes involving in Synaptic Vesicle Cycle mainly distributed in neurons, astrocytes, cycling-T, cycling-Myeloid, macrophage (Figure 7D). So, the neurons might play a pivotal role in reshape TME by regulating crosstalk between tumor cell, immune cell and stromal cell.

**FIGURE 8:**
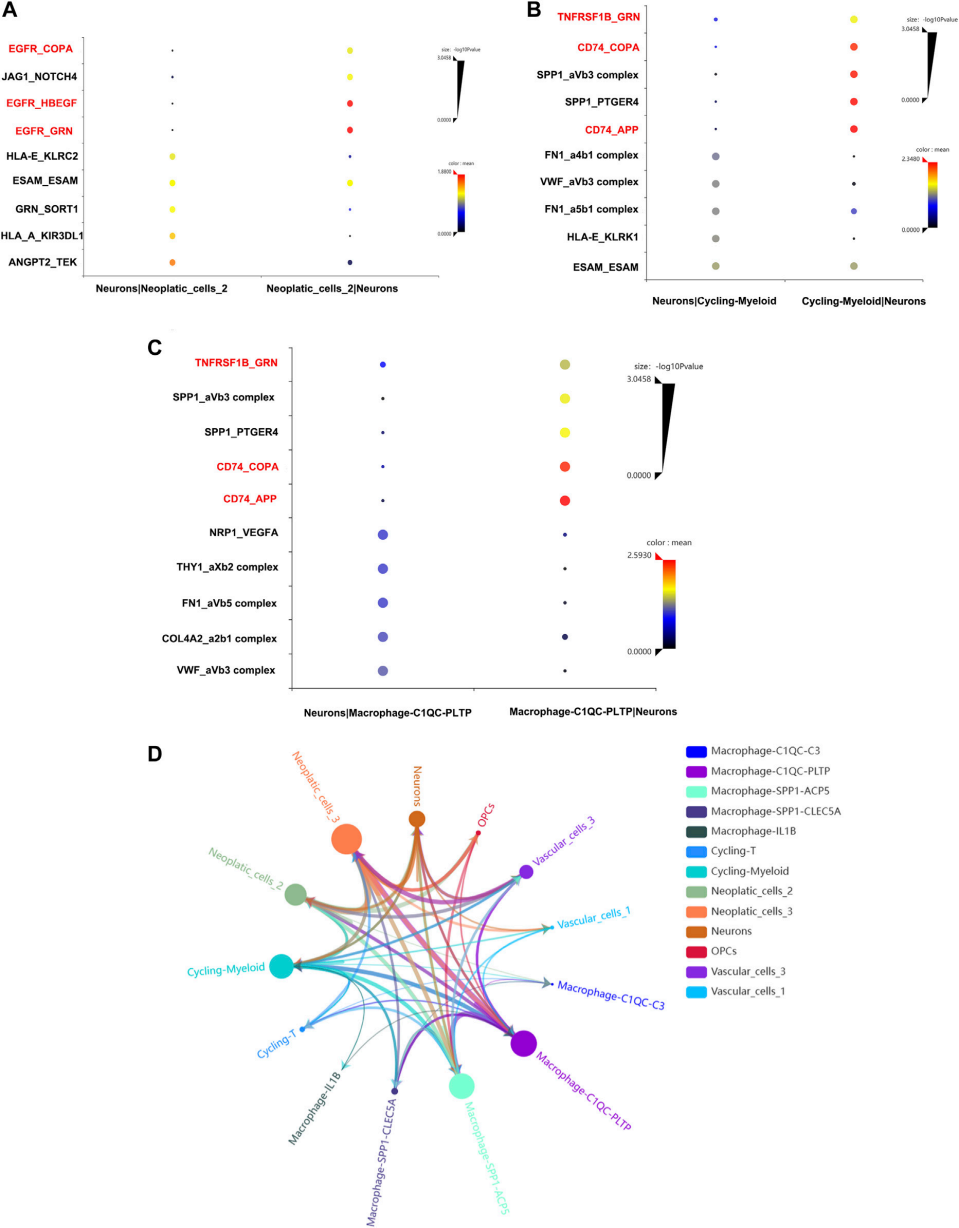
Cell-to-cell communication analysis. **(A)** The ligands and receptors interaction between neurons and neoplastic. **(B)** The ligands and receptors interaction between neurons and cycling-myeloid. **(C)** The ligands and receptors interaction between neurons and macrophages. **(D)** The cell-to-cell communication between all cell types.

To confirmed our conjecture, cell-to-cell communication analysis was conducted. The ligand and receptor pair strength was shown in Figures 8A–C. We noticed that EGFR gene plays important role in the communication between neurons and neoplastic cells (Figure 8A). Additionally, neurons and cycling-myeloid, neurons and macrophage also exhibit communications. Surprisingly, EGFR, COPA, GRN, TNFRSF1B were key ligands and receptors participant in communications between neurons, macrophage and neopaltic cells. So, we confirmed the key role of EGFR again, which may play an important role in regulating the crosstalk between neurons and TME. The communications between all cells were shown in Figure 8D.

### The effects of EGFR and EGFR mutation on immune function

**FIGURE 9:**
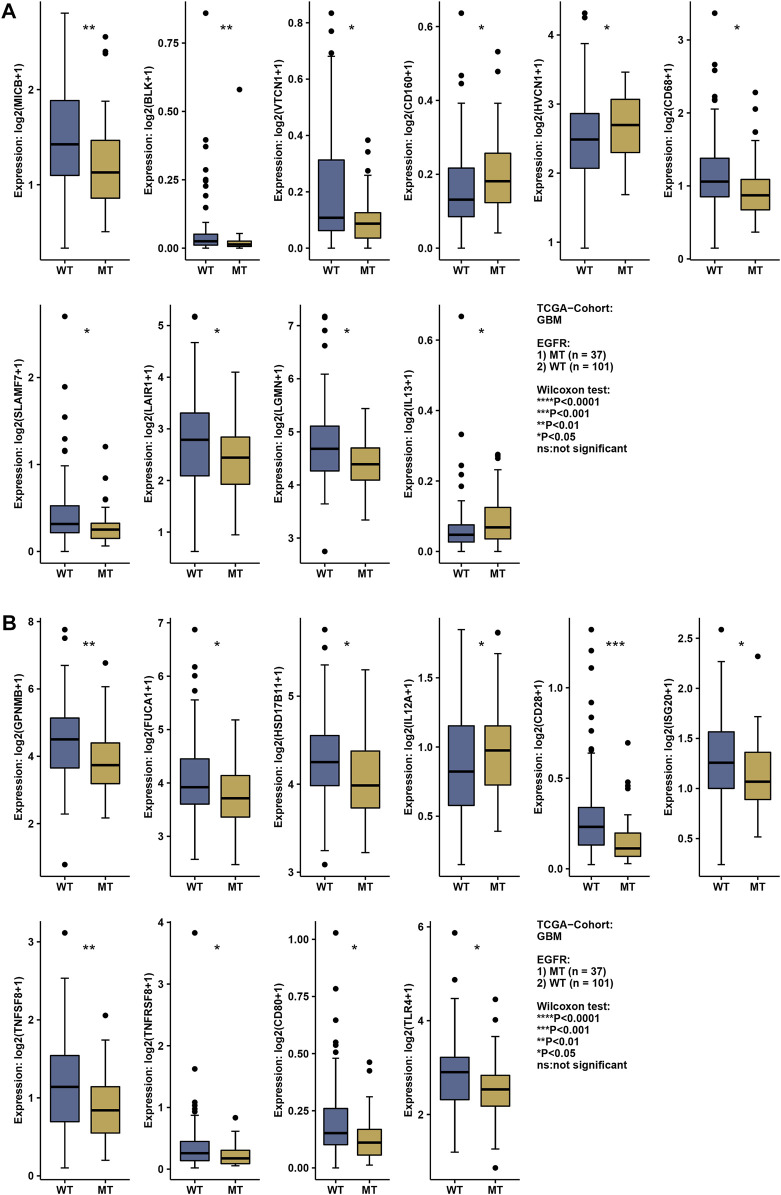
The expression level of the immune-related gene in EGFR wildtype and mutation patients **(A,B)**. * represents a significant difference (*p* < 0.05), ** represents a significant difference (*p* < 0.01), *** represents a significant difference (*p* < 0.001).

**FIGURE 10:**
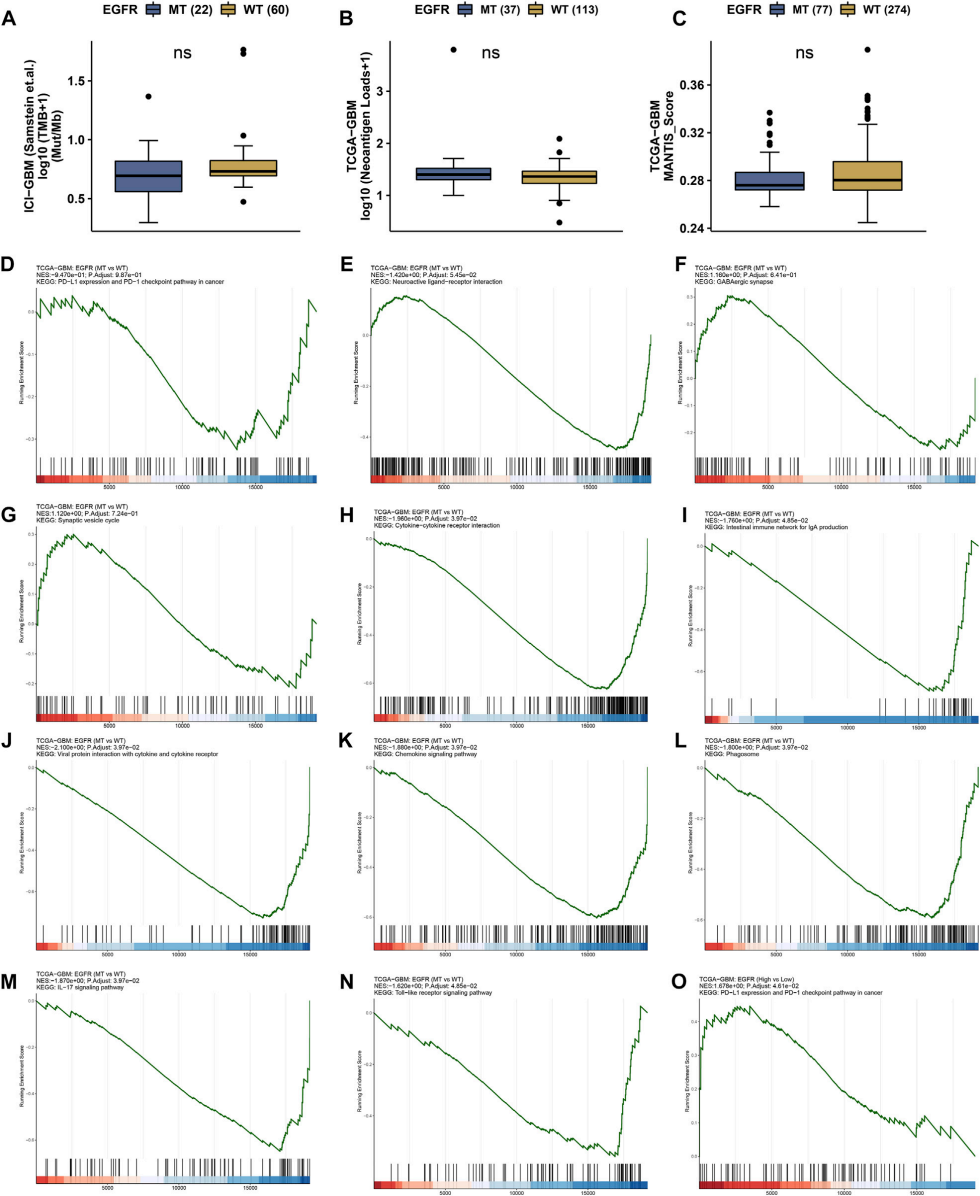
Immune landscape in EGFR wildtype and mutation patients. **(A)** Tumor mutation burden (TMB) status in EGFR wildtype and mutation groups. **(B)** Neoantigen status in EGFR wildtype and mutation groups. **(C)** MANTIS_Score in EGFR wildtype and mutation groups. ns represents no significant difference. **(D–O)** GSEA results in TCGA-GBM cohort.

The status of EGFR plays a dominant role in the therapeutic effect, which might attribute to the immune function difference. Here, we explore the landscape of immune genes in TCGA-GBM cohort (Figures 9A, B). We noticed that CD160, HVCN1, IL13, IL12A were upregulated in EGFR mutated samples. However, EGFR status might not affect the aspect of immunogenicity in GBM (Figures 10A–C). Additionally, the pathways including PD-L1 expression and PD-1 checkpoint pathway, Neuroactive ligand−receptor interaction, GABAergic Synapse and Synaptic vesicle cycle showed no difference between the EGFR-mutated patients and EGFR-wildtype patients (Figures 10D–G). Interestingly, we noticed that some immune-related pathways were downregulated in the EGFR-mutated group, including Cytokine-cytokine receptor interaction, Intestinal immune network for IgA production, Viral protein interaction with cytokine and cytokine receptor, Chemokine signaling pathway, Phagosome, IL-17 signaling pathway and Toll-like receptor signaling pathway (Figures 10H–N). So, EGFR-mutated patients might suffer a worse immune status. In addition to mutation status, EGFR expression also affects immune checkpoint-related pathways. The PD-L1 expression and PD-1 checkpoint pathway was upregulated in the patients with high EGFR expression level (Figure 10O). In conclusion, if imipramine can target EGFR and EGFR mutants, it may improve the immune microenvironment of patients.

### Molecular docking

**FIGURE 11:**
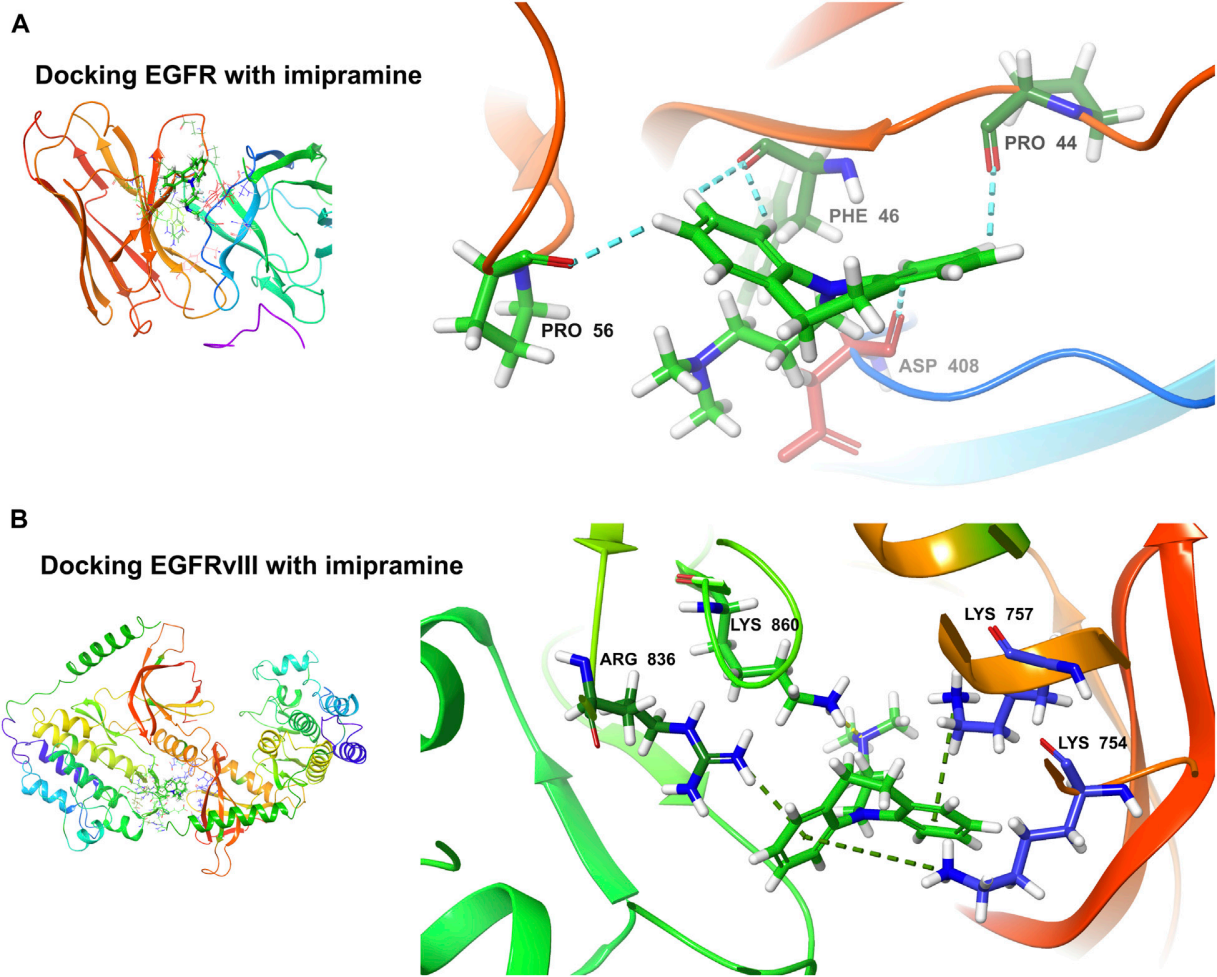
Molecular docking. **(A)** Docking EGFR with imipramine (−4.291 kcal/mol). **(B)** Docking EGFRvIII with imipramine (−5.242 kcal/mol).

The binding ability of the key targets was predicted using molecular docking technology. According to the general consensus, binding energies below −4.25 kcal/mol indicate specific binding between the ligand and the receptor. A binding energy below −5.0 kcal/mol indicates enhanced binding capability. Through molecular docking, we found that both EGFR (−4.291 kcal/mol) and EGFRvIII (−5.242 kcal/mol) have an excellent binding ability to the imipramine (Figures 11A, B).

### Root-mean-square deviation (RMSD) analyses

**FIGURE 12:**
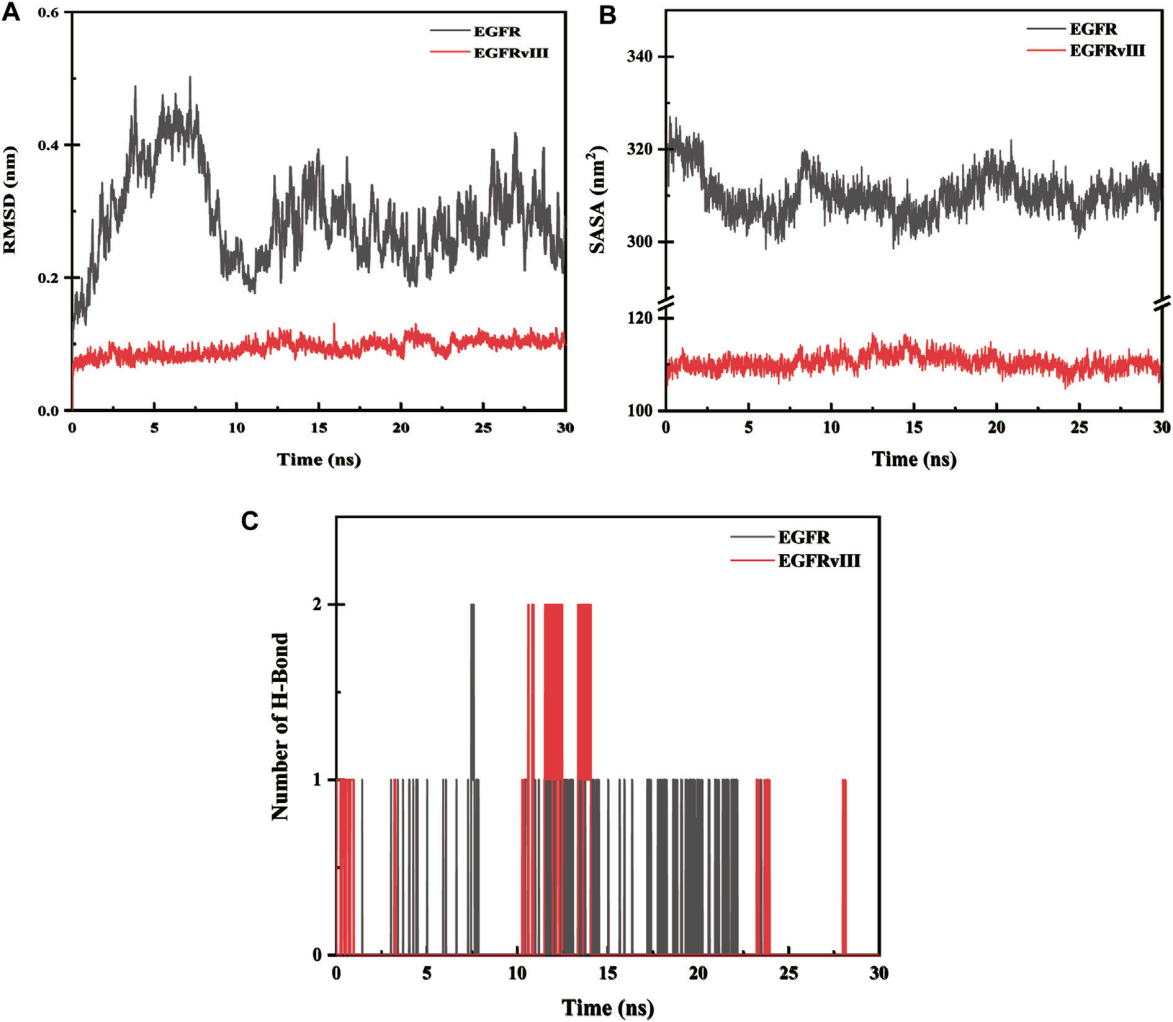
**(A)** Root-mean-square deviation (RMSD) analysis. **(B)** The solvent-accessible surface areas. **(C)** Hydrogen bond interaction.

The root-mean-square deviation (abbreviated as RMSD) is a measure of the deviation of atomic coordinates relative to a reference structure, and it is often used to evaluate whether a simulation system has reached stability. A stable RMSD indicates that the corresponding atoms have become stable, while a fluctuating RMSD implies fluctuations. The RMSD value of EGFR-imipramine is higher than that of EGFRvIII-imipramine, which indicates that the stability of the EGFRvIII-imipramine composite system is higher than that of the EGFR-imipramine composite system (Figure 12A).

### The solvent-accessible surface areas

The solvent accessible surface area (SASA) is calculated by considering the interactions between van der Waals forces and solvent molecules on the solute surface. The SASA of a protein decreases as the compactness of the protein increases, so changes in SASA can be used to predict alterations in protein structure. The fluctuations in SASA values for EGFR-imipramine are greater than those for EGFRvIII-imipramine, indicating that the stability of the EGFRvIII-imipramine composite system is higher than that of the EGFR-imipramine composite system (Figure 12B).

### Hydrogen bond interaction

To investigate the interactions between proteins and ligands, we first performed a hydrogen bond analysis on the protein-ligand complexes. We found that the average number of hydrogen bonds for EGFR-imipramine and EGFRvIII-imipramine were 0.09 and 0.13, respectively. This indicates that the hydrogen bond interaction strength between EGFRvIII and imipramine is greater than that between EGFR and imipramine (Figure 12C).

### MMPBSA the free energy

To better explain the interaction energy between ligands and receptors, we used the gmx_mmpbsa script (https://jerkwin.github.io/gmxtool/) to determine the binding energies of all protein-ligand complexes during the equilibrium phase. In the application of the MMPBSA method, the total binding energy is decomposed into four independent parts (electrostatic interactions, van der Waals interactions, and polar and nonpolar solvation interactions). The results of the protein-ligand binding energies are shown in Table 1. In the protein-ligand complex systems, the main interaction energies are van der Waals and electrostatic energy. The binding free energies of EGFR and EGFRvIII proteins with imipramine are −55.882 and −64.786 kJ/mol, respectively, further indicating that the interaction strength between EGFRvIII and imipramine is greater than that between EGFR and imipramine.

**TABLE 1:** Analysis of MMPBSA.

Energy	EGFR - imipramine	EGFRvIII - imipramine
Van der Waals Energy (KJ/mol)	−98.372	−134.599
Electrostatic energy (KJ/mol)	−29.479	−26.148
Polar solvation energy (KJ/mol)	73.338	99.033
Nonpolar solvation Energy (KJ/mol)	−15.879	−17.653
Total Binding Energy (KJ/mol)	−70.392	−79.368
T∆S(KJ/mol)	14.510	14.582
Total Binding Free Energy (KJ/mol)	−55.882	−64.786

## Conclusion

Many studies have focused on imipramine acting directly on tumor cells to exert a therapeutic effect. However, the therapeutic mechanism of imipramine exerted on other cells in the GBM tumor microenvironment has not been fully elucidated. In this study, two main hypotheses with clinical significance were established. First, based on network pharmacology, single-cell sequencing and molecular docking, we identified neuron-derived EGFR as a possible key target of imipramine. In this way, imipramine might play a key role in regulating crosstalk between neurons, circulating myeloid cells, and macrophages by target neuron-derived EGFR. Next, since EGFR mutations play an important role in the treatment and drug resistance of GBM, we compared the differences in immune-related genes in wild-type EGFR and mutant EGFR samples to confirm whether the status of EGFR affects the immune system, thus supporting the regulation of EGFR on immune cells mentioned above. We found differences in the expression of immune-related genes between wild-type EGFR patients and mutant EGFR patients. GSEA analysis showed that the immune-related pathways were significantly down-regulated in patients with EGFR mutations, while the PD1 pathway was significantly up-regulated in patients with high EGFR expression. Therefore, if imipramine can be used to simultaneously target EGFR mutants and inhibit the expression level of EGFR, it will be a promising therapeutic strategy.

Since EGFRvIII has pivotal clinical guiding significance in the diagnosis and treatment of GBM, we continued to explore the binding ability of imipramine and EGFRvIII. We found that the binding ability of EGFRvIII and imipramine is stronger. Combined with previous GSEA analysis of mutant EGFR *versus* wild-type EGFR, this finding demonstrates that imipramine may target EGFRvIII, thereby altering its function, which may benefit immune microenvironment, such as downregulation of PD1 and PDL1. Last, whether EGFRvIII derived from extrachromosomal DNA can be bound and blocked by imipramine may be an interesting question (Li et al., 2022).

## Perspective

In recent years, more and more intersections between neuroscience and glioma biology were revealed. Venkataramani et al. have demonstrated that some glioma cells are capable of forming functional synapses with nearby neurons. In addition, these glioma cells with functional synapses are able to form electrically active tissues that can signal to other cells as well to stimulate glioma cells’ migration and growth (Venkataramani et al., 2019). Targeting specific types of postsynaptic signal processing processes, or synapse formation mechanisms might be a promising therapeutic approach. Frank et al. found that the invasion of glioblastoma cells is controlled by a variety of neuronal mechanisms (Venkataramani et al., 2022). Consistently, Monje et al. found that the integration of synapses and electrical signals into neural circuits contributes to the progression of gliomas (Venkatesh et al., 2019). Innovative therapeutic advancements will be developed in the convergence between neuroscience and neuro-oncology.

The role of GABA in immune regulation is emerging. GABA, which is transformed from intracellular glutamine by glutamic acid decarboxylases (GAD1/2). GABA transport signaling through two receptors, GABAAR and GABABR. Elevated levels of GABA in late-stage human tumors are inversely associated with prognosis, as are the expressions of GAD1 and GABABR, which are usually co-expressed in cancer cells, creating an autocrine signaling loop (Hanahan and Monje, 2023). Previous studies have shown that GABA-mediated signaling contributes to the maintenance of cancer cell proliferation and immune evasion. Additionally, GABA influences tumor-promoting inflammation, affecting the balance between the two opposing aspects of the immune response to tumors. When GABA signaling is inhibited, this balance shifts towards favoring T cell attack on the tumor. Meanwhile, previous studies uncovered a mechanism for this immune evasion: GABA/GABABR/β-catenin signaling in cancer cells suppresses the expression of pro-inflammatory chemokines CCL4/5 (Huang et al., 2022). Additionally, GABA regulates pro-inflammatory macrophage responses associated with metabolic reprogramming and protein succinylation (Fu et al., 2022). In the future, exploring the role of GABA signaling in maintaining the balance between tumor-promoting inflammation and anti-tumor immune responses will be an intriguing area of study. Moreover, with the development of technologies such as spatial transcriptomics and spatial metabolomics, in some specific brain regions, such as the hippocampus or other regions, neuron-related transcription and metabolic signals will be more deciphered.

## Limitations

The hypothesis proposed in this paper still has limitations, the following are some aspects that need to be further improved. First, due to the limitation of current single-cell sequencing technology, it is difficult to effectively capture a sufficient number of neurons in GBM tissue samples, which is caused by the large cell body and axonal structure of neurons. This poses a barrier to dissecting neuronal-tumor microenvironment communication at the single-cell level. Second, our study has a single-cohort bias, and the differences in the GABA pathway between normal and GBM samples, as well as the differences in immune function between wild-type EGFR and mutant EGFR, need to be verified in large-scale external cohorts. Third, further validation of molecular biology and pharmacology experiments is needed.

## Data Availability

The datasets presented in this study can be found in online repositories. The names of the repository/repositories and accession number(s) can be found in the article/Supplementary Material.

## Supplementary material

Supplementary Figure S1 The batch effect has been removed.
