# Supplementary figures and images for: Will EGFRvIII and neuronal-derived EGFR be targets for imipramine?
Source: Front Pharmacol. 2023 May 30;14:1156492. doi: 10.3389/fphar.2023.1156492 (PMC10266953; doi:10.3389/fphar.2023.1156492)

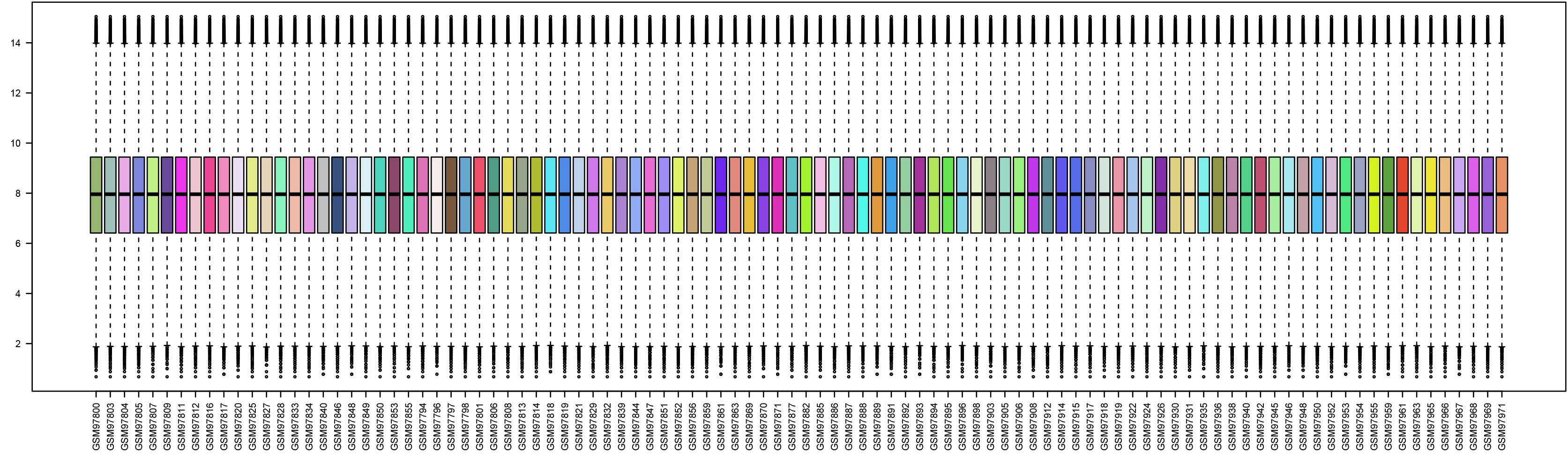

Supplement: Supplementary file 3 [file Image1.TIF]
